# Identifying problems and solutions in scientific text

**DOI:** 10.1007/s11192-018-2718-6

**Published:** 2018-04-06

**Authors:** Kevin Heffernan, Simone Teufel

**Affiliations:** Department of Computer Science and Technology, University of Cambridge, 15 JJ Thomson Avenue, Cambridge, CB3 0FD UK

**Keywords:** Problem-solving patterns, Machine learning, Discourse

## Abstract

Research is often described as a problem-solving activity, and as a result, descriptions of problems and solutions are an essential part of the scientific discourse used to describe research activity. We present an automatic classifier that, given a phrase that may or may not be a description of a scientific problem or a solution, makes a binary decision about problemhood and solutionhood of that phrase. We recast the problem as a supervised machine learning problem, define a set of 15 features correlated with the target categories and use several machine learning algorithms on this task. We also create our own corpus of 2000 positive and negative examples of problems and solutions. We find that we can distinguish problems from non-problems with an accuracy of 82.3%, and solutions from non-solutions with an accuracy of 79.7%. Our three most helpful features for the task are syntactic information (POS tags), document and word embeddings.

## Introduction

Problem solving is generally regarded as the most important cognitive activity in everyday and professional contexts (Jonassen 2000). Many studies on formalising the cognitive process behind problem-solving exist, for instance (Chandrasekaran 1983). Jordan (1980) argues that we all share knowledge of the thought/action problem-solution process involved in real life, and so our writings will often reflect this order. There is general agreement amongst theorists that state that the nature of the research process can be viewed as a problem-solving activity (Strübing 2007; Van Dijk 1980; Hutchins 1977; Grimes 1975).

One of the best-documented problem-solving patterns was established by Winter (1968). Winter analysed thousands of examples of technical texts, and noted that these texts can largely be described in terms of a four-part pattern consisting of Situation, Problem, Solution and Evaluation. This is very similar to the pattern described by Van Dijk (1980), which consists of Introduction-Theory, Problem-Experiment-Comment and Conclusion. The difference is that in Winter’s view, a solution only becomes a solution after it has been evaluated positively. Hoey changes Winter’s pattern by introducing the concept of Response in place of Solution (Hoey 2001). This seems to describe the situation in science better, where evaluation is mandatory for research solutions to be accepted by the community. In Hoey’s pattern, the Situation (which is generally treated as optional) provides background information; the Problem describes an issue which requires attention; the Response provides a way to deal with the issue, and the Evaluation assesses how effective the response is.

**Fig. 1:**
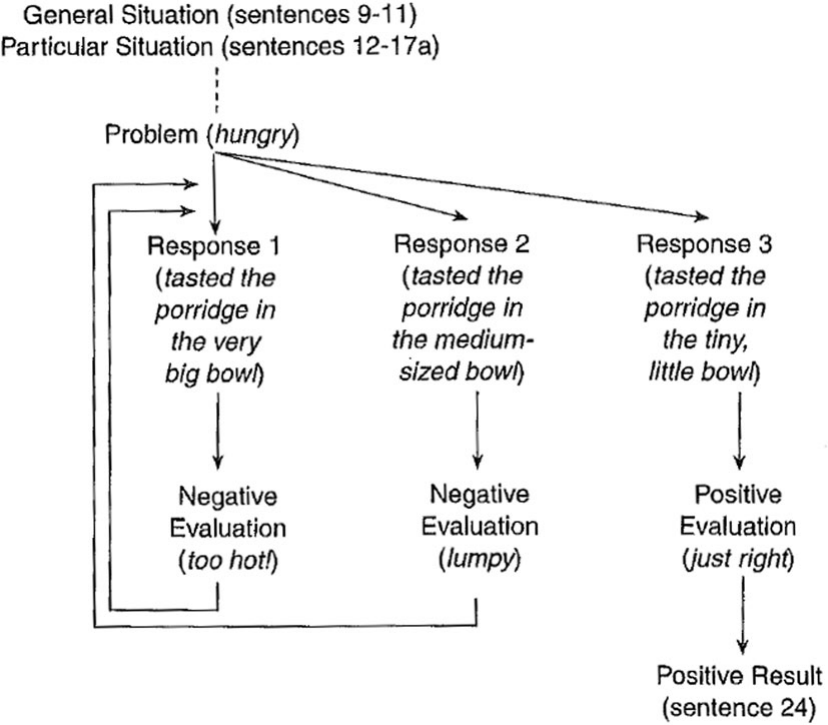
Example of problem-solving pattern when applied to the Goldilocks story. Reproduced with permission from Hoey (2001)

An example of this pattern in the context of the Goldilocks story can be seen in Fig. 1. In this text, there is a preamble providing the setting of the story (i.e. Goldilocks is lost in the woods), which is called the Situation in Hoey’s system. A Problem in encountered when Goldilocks becomes hungry. Her first Response is to try the porridge in big bear’s bowl, but she gives this a negative Evaluation (“too hot!”) and so the pattern returns to the Problem. This continues in a cyclic fashion until the Problem is finally resolved by Goldilocks giving a particular Response a positive Evaluation of baby bear’s porridge (“it’s just right”).

It would be attractive to detect problem and solution statements automatically in text. This holds true both from a theoretical and a practical viewpoint. Theoretically, we know that sentiment detection is related to problem-solving activity, because of the perception that “bad” situations are transformed into “better” ones via problem-solving. The exact mechanism of how this can be detected would advance the state of the art in text understanding. In terms of linguistic realisation, problem and solution statements come in many variants and reformulations, often in the form of positive or negated statements about the conditions, results and causes of problem–solution pairs. Detecting and interpreting those would give us a reasonably objective manner to test a system’s understanding capacity. Practically, being able to detect any mention of a problem is a first step towards detecting a paper’s specific research goal. Being able to do this has been a goal for scientific information retrieval for some time, and if successful, it would improve the effectiveness of scientific search immensely. Detecting problem and solution statements of papers would also enable us to compare similar papers and eventually even lead to automatic generation of review articles in a field.

There has been some computational effort on the task of identifying problem-solving patterns in text. However, most of the prior work has not gone beyond the usage of keyword analysis and some simple contextual examination of the pattern. Flowerdew (2008) presents a corpus-based analysis of lexio-grammatical patterns for problem and solution clauses using articles from professional and student reports. Problem and solution keywords were used to search their corpora, and each occurrence was analysed to determine grammatical usage of the keyword. More interestingly, the causal category associated with each keyword in their context was also analysed. For example, Reason–Result or Means-Purpose were common causal categories found to be associated with problem keywords.

The goal of the work by Scott (2001) was to determine words which are semantically similar to problem and solution, and to determine how these words are used to signal problem-solution patterns. However, their corpus-based analysis used articles from the Guardian newspaper. Since the domain of newspaper text is very different from that of scientific text, we decided not to consider those keywords associated with problem-solving patterns for use in our work.

Instead of a keyword-based approach, Charles (2011) used discourse markers to examine how the problem-solution pattern was signalled in text. In particular, they examined how adverbials associated with a result such as *“thus, therefore, then, hence”* are used to signal a problem-solving pattern.

Problem solving also has been studied in the framework of discourse theories such as Rhetorical Structure Theory (Mann and Thompson 1988) and Argumentative Zoning (Teufel et al. 2000). Problem- and solutionhood constitute two of the original 23 relations in RST (Mann and Thompson 1988). While we concentrate solely on this aspect, RST is a general theory of discourse structure which covers many intentional and informational relations. The relationship to Argumentative Zoning is more complicated. The status of certain statements as problem or solutions is one important dimension in the definitions of AZ categories. AZ additionally models dimensions other than problem-solution hood (such as who a scientific idea belongs to, or which intention the authors might have had in stating a particular negative or positive statement). When forming categories, AZ combines aspects of these dimensions, and “flattens” them out into only 7 categories. In AZ it is crucial *who* it is that experiences the problems or contributes a solution. For instance, the definition of category “CONTRAST” includes statements that some research runs into problems, but only if that research is previous work (i.e., not if it is the work contributed in the paper itself). Similarly, “BASIS” includes statements of successful problem-solving activities, but only if they are achieved by previous work that the current paper bases itself on. Our definition is simpler in that we are interested only in problem solution structure, not in the other dimensions covered in AZ. Our definition is also more far-reaching than AZ, in that we are interested in *all* problems mentioned in the text, no matter whose problems they are. Problem-solution recognition can therefore be seen as one aspect of AZ which can be independently modelled as a “service task”. This means that good problem solution structure recognition should theoretically improve AZ recognition.

In this work, we approach the task of identifying problem-solving patterns in scientific text. We choose to use the model of problem-solving described by Hoey (2001). This pattern comprises four parts: Situation, Problem, Response and Evaluation. The Situation element is considered optional to the pattern, and so our focus centres on the core pattern elements.

## Goal statement and task

Many surface features in the text offer themselves up as potential signals for detecting problem-solving patterns in text. However, since Situation is an optional element, we decided to focus on either Problem or Response and Evaluation as signals of the pattern. Moreover, we decide to look for each type in isolation. Our reasons for this are as follows: It is quite rare for an author to introduce a problem without resolving it using some sort of response, and so this is a good starting point in identifying the pattern. There are exceptions to this, as authors will sometimes introduce a problem and then leave it to future work, but overall there should be enough signal in the Problem element to make our method of looking for it in isolation worthwhile. The second signal we look for is the use of Response and Evaluation within the same sentence. Similar to Problem elements, we hypothesise that this formulation is well enough signalled externally to help us in detecting the pattern. For example, consider the following Response and Evaluation: “One solution is to use smoothing”. In this statement, the author is explicitly stating that smoothing is a solution to a problem which must have been mentioned in a prior statement. In scientific text, we often observe that solutions implicitly contain both Response and Evaluation (positive) elements. Therefore, due to these reasons there should be sufficient external signals for the two pattern elements we concentrate on here.

When attempting to find Problem elements in text, we run into the issue that the word “problem” actually has at least two word senses that need to be distinguished. There is a word sense of “problem” that means something which must be undertaken (i.e. task), while another sense is the core sense of the word, something that is problematic and negative. Only the latter sense is aligned with our sense of problemhood. This is because the simple description of a task does not predispose problemhood, just a wish to perform some act. Consider the following examples, where the non-desired word sense is being used:

- *“Das and Petrov (2011) also consider the problem of unsupervised bilingual POS induction”.* (Chen et al. 2011).
- *“In this paper, we describe advances on the problem of NER in Arabic Wikipedia”.* (Mohit et al. 2012).

Here, the author explicitly states that the phrases in orange are problems, they align with our definition of research tasks and not with what we call here ‘problematic problems’. We will now give some examples from our corpus for the desired, core word sense:

- *“The major limitation of supervised approaches is that they require annotations for example sentences.”* (Poon and Domingos 2009).
- *“To solve the problem of high dimensionality we use clustering to group the words present in the corpus into much smaller number of clusters”.* (Saha et al. 2008).

When creating our corpus of positive and negative examples, we took care to select only problem strings that satisfy our definition of problemhood; “Corpus creation” section will explain how we did that.

## Corpus creation

Our new corpus is a subset of the latest version of the ACL anthology released in March, 2016^1^ which contains 22,878 articles in the form of PDFs and OCRed text.^2^

The 2016 version was also parsed using ParsCit (Councill et al. 2008). ParsCit recognises not only document structure, but also bibliography lists as well as references within running text. A random subset of 2500 papers was collected covering the entire ACL timeline. In order to disregard non-article publications such as introductions to conference proceedings or letters to the editor, only documents containing abstracts were considered. The corpus was preprocessed using tokenisation, lemmatisation and dependency parsing with the Rasp Parser (Briscoe et al. 2006).

### Definition of ground truth

Our goal was to define a ground truth for problem and solution strings, while covering as wide a range as possible of syntactic variations in which such strings naturally occur. We also want this ground truth to cover phenomena of problem and solution status which are applicable whether or not the problem or solution status is explicitly mentioned in the text.

To simplify the task, we only consider here problem and solution descriptions that are at most one sentence long. In reality, of course, many problem descriptions and solution descriptions go beyond single sentence, and require for instance an entire paragraph. However, we also know that short summaries of problems and solutions are very prevalent in science, and also that these tend to occur in the most prominent places in a paper. This is because scientists are trained to express their contribution and the obstacles possibly hindering their success, in an informative, succinct manner. That is the reason why we can afford to only look for shorter problem and solution descriptions, ignoring those that cross sentence boundaries.

**Fig. 2:**
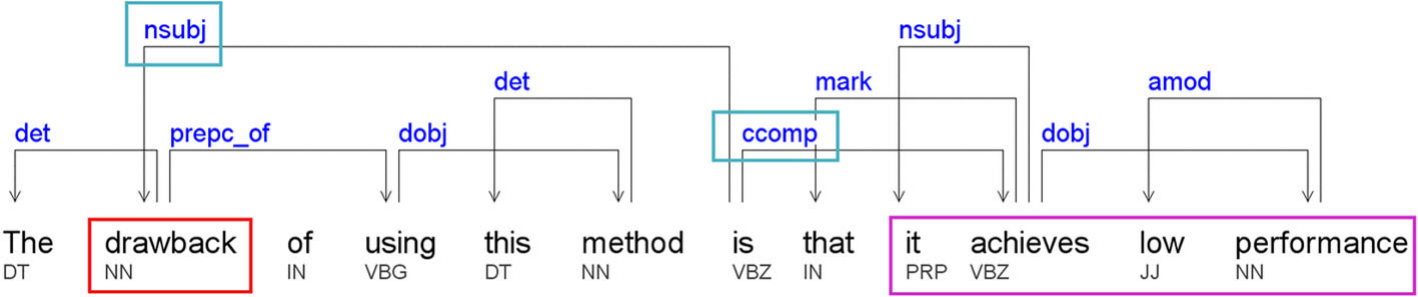
Example of our extraction method for problems using dependencies. (Color figure online)

To define our ground truth, we examined the parsed dependencies and looked for a target word (“problem/solution”) in subject position, and then chose its syntactic argument as our candidate problem or solution phrase. To increase the variation, i.e., to find as many different-worded problem and solution descriptions as possible, we additionally used semantically similar words (near-synonyms) of the target words “problem” or “solution” for the search. Semantic similarity was defined as cosine in a deep learning distributional vector space, trained using Word2Vec (Mikolov et al. 2013) on 18,753,472 sentences from a biomedical corpus based on all full-text Pubmed articles (McKeown et al. 2016). From the 200 words which were semantically closest to “problem”, we manually selected 28 clear synonyms. These are listed in Table 1. From the 200 semantically closest words to “solution” we similarly chose 19 (Table 2). Of the sentences matching our dependency search, a subset of problem and solution candidate sentences were randomly selected.

**Table 1:** Selected words for use in problem candidate phrase extraction

Bottleneck	Caveat	Challenge	Complication	Conundrum	Difficulty
Flaw	Impediment	Issue	Limitation	Mistake	Obstacle
Riddle	Shortcoming	Struggle	Subproblem	Threat	Tragedy
Dilemma	Disadvantage	Drawback	Fault	Pitfall	Problem
Uncertainty	Weakness	Trouble	Quandary

**Table 2:** Selected words for use in solution candidate phrase extraction

Solution	Alternative	Suggestion	Idea	Way	Proposal
Technique	Remedy	Task	Step	Answer	Approach
Approaches	Strategy	Method	Methodology	Scheme	Answers
Workaround					

An example of this is shown in Fig. 2. Here, the target word “drawback” is in subject position (highlighted in red), and its clausal argument (ccomp) is “(that) it achieves low performance” (highlighted in purple). Examples of other arguments we searched for included copula constructions and direct/indirect objects.

If more than one candidate was found in a sentence, one was chosen at random. Non-grammatical sentences were excluded; these might appear in the corpus as a result of its source being OCRed text.

800 candidates phrases expressing problems and solutions were automatically extracted (1600 total) and then independently checked for correctness by two annotators (the two authors of this paper). Both authors found the task simple and straightforward. Correctness was defined by two criteria:

- The sentence must unambiguously and clearly state the phrase’s status as either a problem or a solution. For problems, the guidelines state that the phrase has to represent one of the following:
  1. An unexplained phenomenon or a problematic state in science; or
  2. A research question; or
  3. An artifact that does not fulfil its stated specification.
  For solutions, the phrase had to represent a response to a problem with a positive evaluation. Implicit solutions were also allowed.
- The phrase must not lexically give away its status as problem or solution phrase.

The second criterion saves us from machine learning cues that are too obvious. If for instance, the phrase itself contained the words “lack of” or “problematic” or “drawback”, our manual check rejected it, because it would be too easy for the machine learner to learn such cues, at the expense of many other, more generally occurring cues.

### Sampling of negative examples

We next needed to find negative examples for both cases. We wanted them not to stand out on the surface as negative examples, so we chose them so as to mimic the obvious characteristics of the positive examples as closely as possible. We call the negative examples ‘non-problems’ and ‘non-solutions’ respectively. We wanted the only differences between problems and non-problems to be of a semantic nature, nothing that could be read off on the surface. We therefore sampled a population of phrases that obey the same statistical distribution as our problem and solution strings while making sure they really are negative examples. We started from sentences *not* containing any problem/solution words (i.e. those used as target words). From each such sentence, we at random selected one syntactic subtree contained in it. From these, we randomly selected a subset of negative examples of problems and solutions that satisfy the following conditions:

- The distribution of the head POS tags of the negative strings should perfectly match the head POS tags^3^ of the positive strings. This has the purpose of achieving the same proportion of surface syntactic constructions as observed in the positive cases.
- The average lengths of the negative strings must be within a tolerance of the average length of their respective positive candidates e.g., non-solutions must have an average length very similar (i.e. $+/-$ small tolerance) to solutions. We chose a tolerance value of 3 characters.

Again, a human quality check was performed on non-problems and non-solutions. For each candidate non-problem statement, the candidate was accepted if it did not contain a phenomenon, a problematic state, a research question or a non-functioning artefact. If the string expressed a research task, without explicit statement that there was anything problematic about it (i.e., the ‘wrong’ sense of “problem”, as described above), it was allowed as a non-problem. A clause was confirmed as a non-solution if the string did not represent both a response and positive evaluation.

If the annotator found that the sentence had been slightly mis-parsed, but did contain a candidate, they were allowed to move the boundaries for the candidate clause. This resulted in cleaner text, e.g., in the frequent case of coordination, when non-relevant constituents could be removed.

From the set of sentences which passed the quality-test for both independent assessors, 500 instances of positive and negative problems/solutions were randomly chosen (i.e. 2000 instances in total). When checking for correctness we found that most of the automatically extracted phrases which did not pass the quality test for problem-/solution-hood were either due to obvious learning cues or instances where the sense of problem-hood used is relating to tasks (cf. “Goal statement and task” section).

## Method

### Experimental design

In our experiments, we used three classifiers, namely Naïve Bayes, Logistic Regression and a Support Vector Machine. For all classifiers an implementation from the WEKA machine learning library (Hall et al. 2009) was chosen. Given that our dataset is small, tenfold cross-validation was used instead of a held out test set. All significance tests were conducted using the (two-tailed) Sign Test (Siegel 1956).

### Linguistic correlates of problem- and solution-hood

We first define a set of features without taking the phrase’s context into account. This will tell us about the disambiguation ability of the problem/solution description’s semantics alone. In particular, we cut out the rest of the sentence other than the phrase and never use it for classification. This is done for similar reasons to excluding certain ‘give-away’ phrases *inside* the phrases themselves (as explained above). As the phrases were found using templates, we know that the machine learner would simply pick up on the semantics of the template, which always contains a synonym of “problem” or “solution”, thus drowning out the more hidden features hopefully inherent in the semantics of the phrases themselves. If we allowed the machine learner to use these stronger features, it would suffer in its ability to generalise to the real task.

*ngrams* Bags of words are traditionally successfully used for classification tasks in NLP, so we included bags of words (lemmas) within the candidate phrases as one of our features (and treat it as a baseline later on). We also include bigrams and trigrams as multi-word combinations can be indicative of problems and solutions e.g., “combinatorial explosion”.

*Polarity* Our second feature concerns the polarity of each word in the candidate strings. Consider the following example of a problem taken from our dataset: *“very conservative approaches to exact and partial string matches overgenerate badly”.* In this sentence, words such as “badly” will be associated with negative polarity, therefore being useful in determining problem-hood. Similarly, solutions will often be associated with a positive sentiment e.g. *“smoothing is a good way to overcome data sparsity”*. To do this, we perform word sense disambiguation of each word using the Lesk algorithm (Lesk 1986). The polarity of the resulting synset in SentiWordNet (Baccianella et al. 2010) was then looked up and used as a feature.

*Syntax* Next, a set of syntactic features were defined by using the presence of POS tags in each candidate. This feature could be helpful in finding syntactic patterns in problems and solutions. We were careful not to base the model directly on the head POS tag and the length of each candidate phrase, as these are defining characteristics used for determining the non-problem and non-solution candidate set.

*Negation* Negation is an important property that can often greatly affect the polarity of a phrase. For example, a phrase containing a keyword pertinent to solution-hood may be a good indicator but with the presence of negation may flip the polarity to problem-hood e.g., “this can’t work as a solution”. Therefore, presence of negation is determined.

*Exemplification and contrast* Problems and solutions are often found to be coupled with examples as they allow the author to elucidate their point. For instance, consider the following solution: *“Once the translations are generated, an obvious solution is to pick the most fluent alternative, e.g., using an n-gram language model”.* (Madnani et al. 2012). To acknowledge this, we check for presence of exemplification. In addition to examples, problems in particular are often found when contrast is signalled by the author (e.g. “however, “but”), therefore we also check for presence of contrast in the problem and non-problem candidates only.

*Discourse* Problems and solutions have also been found to have a correlation with discourse properties. For example, problem-solving patterns often occur in the background sections of a paper. The rationale behind this is that the author is conventionally asked to objectively criticise other work in the background (e.g. describing research gaps which motivate the current paper). To take this in account, we examine the context of each string and capture the section header under which it is contained (e.g. Introduction, Future work). In addition, problems and solutions are often found following the Situation element in the problem-solving pattern (cf. “Introduction” section). This preamble setting up the problem or solution means that these elements are likely not to be found occurring at the beginning of a section (i.e. it will usually take some sort of introduction to detail how something is problematic and why a solution is needed). Therefore we record the distance from the candidate string to the nearest section header.

*Subcategorisation and adverbials* Solutions often involve an activity (e.g. a task). We also model the subcategorisation properties of the verbs involved. Our intuition was that since problematic situations are often described as non-actions, then these are more likely to be intransitive. Conversely solutions are often actions and are likely to have at least one argument. This feature was calculated by running the C&C parser (Curran et al. 2007) on each sentence. C&C is a supertagger and parser that has access to subcategorisation information. Solutions are also associated with resultative adverbial modification (e.g. “thus, therefore, consequently”) as it expresses the solutionhood relation between the problem and the solution. It has been seen to occur frequently in problem-solving patterns, as studied by Charles (2011). Therefore, we check for presence of resultative adverbial modification in the solution and non-solution candidate only.

*Embeddings* We also wanted to add more information using word embeddings. This was done in two different ways. Firstly, we created a Doc2Vec model (Le and Mikolov 2014), which was trained on $\sim \,19$ million sentences from scientific text (no overlap with our data set). An embedding was created for each candidate sentence. Secondly, word embeddings were calculated using the Word2Vec model (cf. “Corpus creation” section). For each candidate head, the full word embedding was included as a feature. Lastly, when creating our polarity feature we query SentiWordNet using synsets assigned by the Lesk algorithm. However, not all words are assigned a sense by Lesk, so we need to take care when that happens. In those cases, the distributional semantic similarity of the word is compared to two words with a known polarity, namely “poor” and “excellent”. These particular words have traditionally been consistently good indicators of polarity status in many studies (Turney 2002; Mullen and Collier 2004). Semantic similarity was defined as cosine similarity on the embeddings of the Word2Vec model (cf. “Corpus creation” section).

*Modality* Responses to problems in scientific writing often express possibility and necessity, and so have a close connection with modality. Modality can be broken into three main categories, as described by Kratzer (1991), namely epistemic (possibility), deontic (permission / request / wish) and dynamic (expressing ability).

Problems have a strong relationship to modality within scientific writing. Often, this is due to a tactic called “hedging” (Medlock and Briscoe 2007) where the author uses speculative language, often using Epistemic modality, in an attempt to make either noncommital or vague statements. This has the effect of allowing the author to distance themselves from the statement, and is often employed when discussing negative or problematic topics. Consider the following example of Epistemic modality from Nakov and Hearst (2008): *“A potential drawback is that it* **might** *not work well for low-frequency words”.*

To take this linguistic correlate into account as a feature, we replicated a modality classifier as described by (Ruppenhofer and Rehbein 2012). More sophisticated modality classifiers have been recently introduced, for instance using a wide range of features and convolutional neural networks, e.g, (Zhou et al. 2015; Marasović and Frank 2016). However, we wanted to check the effect of a simpler method of modality classification on the final outcome first before investing heavily into their implementation. We trained three classifiers using the subset of features which Ruppenhofer et al. reported as performing best, and evaluated them on the gold standard dataset provided by the authors^4^. The results of the are shown in Table 3. The dataset contains annotations of English modal verbs on the 535 documents of the first MPQA corpus release (Wiebe et al. 2005).

**Table 3:** Modality classifier results (precision/recall/f-measure) using Naïve Bayes (NB), logistic regression, and a support vector machine (SVM)

Modality	Classification accuracy		
	NB	LR	SVM
Epistemic	.74/.74/.74	*.73/.92/.81*	.75/.85/.80
Deontic	.94/.72/.81	*.92/.76/.84*	.86/.81/.83
Dynamic	*.69/.80/.74*	*.70/.79/.74*	.69/.70/.70

Italicized results reflect highest f-measure reported per modal category

Logistic Regression performed best overall and so this model was chosen for our upcoming experiments. With regards to the optative and concessive modal categories, they can be seen to perform extremely poorly, with the optative category receiving a null score across all three classifiers. This is due to a limitation in the dataset, which is unbalanced and contains very few instances of these two categories. This unbalanced data also is the reason behind our decision of reporting results in terms of recall, precision and f-measure in Table 3.

The modality classifier was then retrained on the entirety of the dataset used by Ruppenhofer and Rehbein (2012) using the best performing model from training (Logistic Regression). This new model was then used in the upcoming experiment to predict modality labels for each instance in our dataset.

## Results

### Problems

**Table 4:** Results distinguishing problems from non-problems using Naïve Bayes (NB), logistic regression (LR) and a support vector machine (SVM)

	Feature sets	Classification accuracy		
		NB	SVM	LR
1	$\hbox {Baseline}_{\mathrm{bow}}$	65.6	67.8	71.0
2	Bigrams	61.3	60.5	59.0
3	Contrast	50.6	50.8	50.5
4	Discourse	60.3	60.2	60.0
5	Doc2vec	72.9*	72.7	72.3
6	Exemplification	50.3	50.2	50.0
7	Modality	52.3	52.3	50.3
8	Negation	59.9	59.9	59.9
9	Polarity	60.2	66.3	65.5
10	Syntax	73.6*	76.2**	74.4
11	Subcategorisation	46.9	47.3	49.1
12	Trigrams	57.7	51.2	54.0
13	$\hbox {Word2vec}_{\mathrm{head}}$	57.9	64.1	64.7
14	$\hbox {Word2vec}_{\mathrm{polarity}}$	76.2***	77.2**	76.6
15	All features	79.3***	81.8***	79.3**
16	All features-{2,3,7,12}	79.4***	82.3***	79.0**

Each feature set’s performance is shown in isolation followed by combinations with other features. Tenfold stratified cross-validation was used across all experiments. Statistical significance with respect to the baseline at the $p < 0.05$, 0.01, 0.001 levels is denoted by *, ** and *** respectively

**Table 5:** Information gain (IG) in bits of top lemmas from the bag-of-words baseline in Table 4

IG	Features
0.048	Not
0.019	Do
0.018	Single
0.013	Limited, experiment
0.010	Data, information
0.009	Error, many
0.008	Take, explosion

As can be seen from Table 4, we are able to achieve good results for distinguishing a problematic statement from non-problematic one. The bag-of-words baseline achieves a very good performance of 71.0% for the Logistic Regression classifier, showing that there is enough signal in the candidate phrases alone to distinguish them much better than random chance.

Taking a look at Table 5, which shows the information gain for the top lemmas,

we can see that the top lemmas are indeed indicative of problemhood (e.g. “limit”,“explosion”). Bigrams achieved good performance on their own (as did negation and discourse) but unfortunately performance deteriorated when using trigrams, particularly with the SVM and LR. The subcategorisation feature was the worst performing feature in isolation. Upon taking a closer look at our data, we saw that our hypothesis that intransitive verbs are commonly used in problematic statements was true, with over 30% of our problems (153) using them. However, due to our sampling method for the negative cases we also picked up many intransitive verbs (163). This explains the almost random chance performance (i.e.  50%) given that the distribution of intransitive verbs amongst the positive and negative candidates was almost even.

The modality feature was the most expensive to produce, but also didn’t perform very well is isolation. This surprising result may be partly due to a data sparsity issue

where only a small portion (169) of our instances contained modal verbs. The breakdown of how many types of modal senses which occurred is displayed in Table 6. The most dominant modal sense was epistemic. This is a good indicator of problemhood (e.g. hedging, cf. “Linguistic correlates of problem- and solution-hood” section) but if the accumulation of additional data was possible, we think that this feature may have the potential to be much more valuable in determining problemhood. Another reason for the performance may be domain dependence of the classifier since it was trained on text from different domains (e.g. news). Additionally, modality has also shown to be helpful in determining contextual polarity (Wilson et al. 2005) and argumentation (Becker et al. 2016), so using the output from this modality classifier may also prove useful for further feature engineering taking this into account in future work.

**Table 6:** Number of instances of modal senses

	No. of instances
Epistemic	97
Deontic	22
Dynamic	50

Polarity managed to perform well but not as good as we hoped. However, this feature also suffers from a sparsity issue resulting from cases where the Lesk algorithm (Lesk 1986) is not able to resolve the synset of the syntactic head.

**Table 7:** Confusion matrix for problems

	Predicted	
	Problem	Non-problem
Actual		
Problem	414	86
Non-problem	91	409

Knowledge of syntax provides a big improvement with a significant increase over the baseline results from two of the classifiers.

Examining this in greater detail, POS tags with high information gain mostly included tags from open classes (i.e. VB-, JJ-, NN- and RB-). These tags are often more associated with determining polarity status than tags such as prepositions and conjunctions (i.e. adverbs and adjectives are more likely to be describing something with a non-neutral viewpoint).

The embeddings from Doc2Vec allowed us to obtain another significant increase in performance (72.9% with Naïve Bayes) over the baseline and polarity using Word2Vec provided the best individual feature result (77.2% with SVM).

Combining all features together, each classifier managed to achieve a significant result over the baseline with the best result coming from the SVM (81.8%). Problems were also better classified than non-problems as shown in the confusion matrix in Table 7. The addition of the Word2Vec vectors may be seen as a form of smoothing in cases where previous linguistic features had a sparsity issue i.e., instead of a NULL entry, the embeddings provide some sort of value for each candidate. Particularly wrt. the polarity feature, cases where Lesk was unable to resolve a synset meant that a ZERO entry was added to the vector supplied to the machine learner. Amongst the possible combinations, the best subset of features was found by combining all features with the exception of bigrams, trigrams, subcategorisation and modality. This subset of features managed to improve results in both the Naïve Bayes and SVM classifiers with the highest overall result coming from the SVM (82.3%).

### Solutions

**Table 8:** Results distinguishing solutions from non-solutions using Naïve Bayes (NB), logistic regression (LR) and a support vector machine (SVM)

	Feature sets	Classification accuracy		
		NB	SVM	LR
1	$\hbox {Baseline}_{\mathrm{bow}}$	72.5	73.6	70.7
2	Adverbial of result	48.3	50.5	50.3
3	Bigrams	63.1	65.1	59.8
4	Discourse	56.9	56.4	58.2
5	Doc2vec	65.9	68.7	67.7
6	Exemplification	63.1	65.1	59.8
7	Negation	63.1	65.1	59.8
8	Polarity	63.1	65.1	59.8
9	Subcategorisation	55.4	53.3	55.3
10	Syntax	61.9	62.2	64.4
11	Trigrams	63.1	65.1	59.8
12	$\hbox {Word2vec}_{\mathrm{head}}$	68.2	70.7	68.9
13	$\hbox {Word2vec}_{\mathrm{polarity}}$	72.1	73.4	69.4
14	All features	*74.5*	79.5	73.1
15	All features-{2,3,6,7,8,13}	73.8	*79.7*	*74.3*

Each feature set’s performance is shown in isolation followed by combinations with other features. Tenfold stratified cross-validation was used across all experiments

**Table 9:** Information gain (IG) in bits of top lemmas from the bag-of-words baseline in Table 8

IG	Features
0.076	Be
0.047	Use
0.014	Method
0.013	Argument
0.012	Dependency
0.011	Configuration, sequence, subject
0.009	Label, weakest
0.008	Following, edge, employ

The results for disambiguation of solutions from non-solutions can be seen in Table 8. The bag-of-words baseline performs much better than random, with the performance being quite high with regard to the SVM (this result was also higher than any of the baseline performances from the problem classifiers). As shown in Table 9, the top ranked lemmas from the best performing model (using information gain) included “use” and “method”. These lemmas are very indicative of solutionhood and so give some insight into the high baseline returned from the machine learners. Subcategorisation and the result adverbials were the two worst performing features. However, the low performance for subcategorisation is due to the sampling of the non-solutions (the same reason for the low performance of the problem transitivity feature). When fitting the POS-tag distribution for the negative samples, we noticed that over 80% of the head POS-tags were verbs (much higher than the problem heads). The most frequent verb type being the infinite form.

**Table 10:** Confusion matrix for solutions

	Predicted	
	Solution	Non-solution
Actual		
Solution	411	89
Non-solution	114	386

This is not surprising given that a very common formulation to describe a solution is to use the infinitive “TO” since it often describes a task e.g., *“One solution is to find the singletons and remove them”.* Therefore, since the head POS tags of the non-solutions had to match this high distribution of infinitive verbs present in the solution, the subcategorisation feature is not particularly discriminatory. Polarity, negation, exemplification and syntactic features were slightly more discriminate and provided comparable results. However, similar to the problem experiment, the embeddings from Word2Vec and Doc2Vec proved to be the best features, with polarity using Word2Vec providing the best individual result (73.4% with SVM).

Combining all features together managed to improve over each feature in isolation and beat the baseline using all three classifiers. Furthermore, when looking at the confusion matrix in Table 10 the solutions were classified more accurately than the non-solutions. The best subset of features was found by combining all features without adverbial of result, bigrams, exemplification, negation, polarity and subcategorisation. The best result using this subset of features was achieved by the SVM with 79.7%. It managed to greatly improve upon the baseline but was just shy of achieving statistical significance ($p=0.057$).

## Discussion

In this work, we have presented new supervised classifiers for the task of identifying problem and solution statements in scientific text. We have also introduced a new corpus for this task and used it for evaluating our classifiers. Great care was taken in constructing the corpus by ensuring that the negative and positive samples were closely matched in terms of syntactic shape. If we had simply selected random subtrees for negative samples without regard for any syntactic similarity with our positive samples, the machine learner may have found easy signals such as sentence length. Additionally, since we did not allow the machine learner to see the surroundings of the candidate string within the sentence, this made our task even harder. Our performance on the corpus shows promise for this task, and proves that there are strong signals for determining both the problem and solution parts of the problem-solving pattern independently.

With regard to classifying problems from non-problems, features such as the POS tag, document and word embeddings provide the best features, with polarity using the Word2Vec embeddings achieving the highest feature performance. The best overall result was achieved using an SVM with a subset of features (82.3%). Classifying solutions from non-solutions also performs well using the embedding features, with the best feature also being polarity using the Word2Vec embeddings, and the highest result also coming from the SVM with a feature subset (79.7%).

In future work, we plan to link problem and solution statements which were found independently during our corpus creation. Given that our classifiers were trained on data solely from the ACL anthology, we also hope to investigate the domain specificity of our classifiers and see how well they can generalise to domains other than ACL (e.g. bioinformatics). Since we took great care at removing the knowledge our classifiers have of the explicit statements of problem and solution (i.e. the classifiers were trained only on the syntactic argument of the explicit statement of problem-/solution-hood), our classifiers should in principle be in a good position to generalise, i.e., find implicit statements too. In future work, we will measure to which degree this is the case.

To facilitate further research on this topic, all code and data used in our experiments can be found here: www.cl.cam.ac.uk/~kh562/identifying-problems-and-solutions.html

## Footnotes

1 http://acl-arc.comp.nus.edu.sg/.

2 The corpus comprises 3,391,198 sentences, 71,149,169 words and 451,996,332 characters.

3 The head POS tags were found using a modification of the Collins’ Head Finder. This modified algorithm addresses some of the limitations of the head finding heuristics described by Collins (2003) and can be found here: http://nlp.stanford.edu/nlp/javadoc/javanlp/edu/stanford/nlp/trees/ModCollinsHeadFinder.html.

4
https://www.uni-hildesheim.de/ruppenhofer/data/modalia_release1.0.tgz.
